# No Gentleman Keeps a Bull Dog

**Published:** 1890-10-04

**Authors:** W. Percy Laing

**Affiliations:** Member of the B. D. C. Brooklands, Romford


### "NO GENTLEMAN KEEPS A BULL DOG."

Dear Sir,—I cannot allow your annotation to go unchallenged, and so hope you will in all fairness insert this letter.

I have nothing to say about prize fighters, but must take up my pen in defence of the Bull Dog and his owner, and as far as they are concerned, deny every word written in that article.

The writer says: "No gentleman keeps a Bull Dog." Will he kindly write to the Hon. Secretary of the Bull Dog Club for a list of its members and he will there find the names of many *Gentlemen* of culture, refinement, and position, who give much of their time and money in breeding and rearing the Englishman's true friend, "the British Bull Dog."

I don't really know whether they have been gauged "by the Laws of Science" (whatever they may be), but I mean gentlemen by birth and education.

Your writer says, "We cannot help sickening a little when we witness a fight between two bull dogs. Has he ever seen one? If so, he has broken the law by aiding that which is illegal. And now, sir, a word for the poor dog itself.

Instead of the Bull Dog being "a typical example of unmitigated savagery and blood lust," I have always found him a most noble example of honesty, pluck, and good temper, a grand houseguard and faithful companion, having far more *morals* (and for that matter, brains), than the writer of your annotation can possibly be credited with.

When the writer in your paper says "both the Bull Dogs and the Prize Fighters might be knocked on the head like hungry wolves," I fancy the public would also gain if those people who write newspaper articles upon things they know *nothing* about were served in the same way.—I am Sir, faithfully yours, W. Percy Laing.

Member of the B. D. C.

Brooklands, Romford, September 29th, 1890.

[The writer of the article has seen, against his will, more than one bull-dog fight in the colliery districts of Yorkshire, where almost every third collier keeps his bull-dog; and a more brutal and sickening exhibition he never witnessed. Moreover, he was once persuaded to keep a bull-dog himself for a short time. He is quite familiar with the ways both of bull-dogs and their owners.—Ed.]

